# Examining an entirely self-guided transdiagnostic internet-delivered intervention ‘in the wild’: A randomised controlled trial for anxiety and depression with minimal inclusion criteria

**DOI:** 10.1016/j.invent.2025.100887

**Published:** 2025-11-02

**Authors:** Blake F. Dear, Alana Fisher, Madelyne Bisby, Amelia Scott, Nickolai Titov

**Affiliations:** a eCentreClinic, School of Psychological Sciences, Macquarie University, Sydney, Australia

**Keywords:** Anxiety, Depression, Internet, CBT, Self-guided, Transdiagnostic

## Abstract

Entirely self-guided transdiagnostic internet-delivered psychological interventions have considerable potential for increasing access to care for adults with anxiety and depression. The current study sought to examine such an intervention under real-world conditions using minimal inclusion and exclusion criteria. Three-hundred and ninety participants were randomised to either an immediate treatment group or a waitlist control group. The intervention consisted of 5 core modules delivered over 8 weeks, according to a structured timetable and with automated emails. Fifty-three percent of participants completed ≥4 of the core modules, and 75 % reported being satisfied with the intervention. At post-treatment, significant improvements were observed in the treatment group compared with control in both anxiety (treatment = 19 % [95 % CI: 12, 26]; control = 8 % [95 % CI: 2, 15]) and depression (treatment = 21 % [95 % CI: 13, 28]; control = 5 % [95 % CI: −1, 12]). These improvements were reflected in significantly greater proportions of participants meeting criteria for clinically meaningful change (≥50 % reduction) in the treatment group for both anxiety (treatment = 25 % [95 % CI: 19, 31]; control = 9 % [95 % CI: 5, 16]) and depression (treatment = 27 % [95 % CI: 20, 36]; control = 8 % [95 % CI: 5, 14]), with numbers needed to treat (NNTs) of 6.6 and 5.3, respectively. The findings of the current study support the potential of entirely self-guided transdiagnostic internet-delivered interventions, particularly for people for whom speaking with a clinician represents a barrier to care, or contexts where the resources for clinician-guided intervention are not available.

## Highlights

• Entirely self-guided and transdiagnostic interventions have public health potential.
• No randomised controlled trials under real-world conditions have been published.
• Relatively high levels of treatment engagement and satisfaction were observed.
• Significant improvements in anxiety and depression were observed over control.

## 1 Introduction

Anxiety and depressive disorders are among the most common mental health problems globally (Kessler et al., 2012), often co-occur (Gorman, 1996) and are associated with significant impacts (Whiteford et al., 2013). Despite their impacts, it is known that many people with these disorders face direct (e.g., financial cost) and indirect barriers (e.g., stigma) in accessing effective care (Mojtabai et al., 2011). This has led to calls for initiatives that can increase access to evidence-based psychological care (Kazdin and Blase, 2011).

One approach to achieving this objective has been the development of internet-delivered psychological interventions (Andersson et al., 2019; Hedman-Lagerlöf et al., 2023). These interventions draw on the same skills and principles as traditional face-to-face therapies, however, they involve carefully developed online modules and learning systems to deliver care without the need for face-to-face contact. These interventions can be delivered with or without clinician support (often termed clinician guided or self-guided, respectively). When offered, clinician support is usually provided by telephone or secure messaging systems alongside the online modules. A large number of studies have demonstrated the clinical effectiveness of internet-delivered interventions, particularly those based on cognitive and behavioural therapies (i.e., internet-delivered CBT [iCBT]) for anxiety and depression (Hedman-Lagerlöf et al., 2023; Andrews et al., 2018; Karyotaki et al., 2021; Karyotaki et al., 2017). For example, a meta-analysis (k = 64; *n* = 8279) of internet-based interventions for several anxiety disorders and depression found evidence of moderate-to-large effects (g = 0.80; 95 % CI: 0.68, 0.92] compared to control (Andrews et al., 2018). A consistent trend in the findings has been that interventions which involve some clinician contact, even if restricted to a pre-treatment assessment phone call, are associated with better outcomes (Krieger et al., 2023). Based on these robust and promising results, several mental health systems have now adopted clinician-guided internet interventions (Etzelmueller et al., 2020; Titov et al., 2018).

Another major development within the field has been the development of transdiagnostic treatments (Cuijpers et al., 2023; Dalgleish et al., 2020). Transdiagnostic treatments support individuals to identify common maintaining factors in their anxiety and depressive symptoms and teach psychological information and skills that the individuals can use to manage their symptoms irrespective of their specific disorders (Dalgleish et al., 2020). Transdiagnostic treatment approaches have significant potential for increasing access to evidence-based care because one treatment protocol can be used with many patients, particularly patients with comorbid conditions, reducing potential demands on services and clinicians while enhancing scalability. Reflecting their potential, numerous trials have examined the outcomes of transdiagnostic internet-delivered interventions for anxiety and depression (Kolaas et al., 2024; Liu et al., 2025). For example, a recent meta-analysis (k = 42; *n* = 4982) found moderate effect sizes for both anxiety (g = 0.58; 95 % CI: 0.47, 0.69) and depression (g = 0.65; 95 % CI: 0.53, 0.77) over control groups, the majority of which were waitlist control (Liu et al., 2025). However, a careful review of the literature reveals that most trials involve some clinician or other human contact, either prior to or during intervention (Kolaas et al., 2024; Liu et al., 2025). To date, very few trials of entirely self-guided (i.e., without any clinician or human contact) transdiagnostic internet interventions for anxiety and depression (Batterham et al., 2021; Titov et al., 2013). This is surprising given the public health potential of combining the benefits of internet-delivery and transdiagnostic approaches with the scalability of an entirely self-guided intervention.

An additional notable limitation of much of the available intervention literature is the tendency of many trials to exclude the types of individuals who seek care in routine practice (Zimmerman et al., 2019; Bisby et al., 2022; Lorenzo-Luaces et al., 2018). For example, the vast majority of trials of internet-delivered and transdiagnostic interventions for anxiety and depression (Kolaas et al., 2024; Liu et al., 2025) include criteria requiring certain levels of symptoms, specific diagnostic criteria, being on a stable dose of medication, not being involved in concurrent psychological treatment, not reporting significant comorbid conditions, not experiencing severe symptoms, and not having suicide-related thoughts or plans. These criteria are often necessary when first evaluating the efficacy of new interventions, where they can help to isolate genuine intervention effects while also helping to ensure the safety of participants. However, such criteria often mean that the characteristics of participants in clinical trials rarely reflect those of people seeking treatment (Wells, 1999; Rothwell, 2005). Moreover, such differences may explain the smaller effects often observed in evaluations of interventions in routine care compared with highly controlled trials. Thus, as the field progresses, there is significant value in broadening inclusion criteria for participation in trials to generate more accurate estimates of the likely real-world utility of new treatment approaches.

In this context, the aim of the current trial was to evaluate the real-world effectiveness of an entirely self-guided, internet-delivered transdiagnostic intervention for adults with anxiety and depression. The trial aimed to examine the intervention under real-world conditions and thus imposed minimal inclusion and exclusion criteria. The trial sought to examine overall effectiveness as well as effectiveness for those with clinical level symptoms and for those who completed the intervention.

## 2 Method

### 2.1 Participants

Participants were recruited via the website of a specialist research clinic (www.ecentreclinic.org), which provides access to free treatment via participation in trials. The clinic is promoted via the websites of numerous governmental and non-governmental organisations, as well as via word-of-mouth recommendations of health professionals and other community groups. The current trial was also promoted via the clinic's social media.

Interested people could read about the intervention and the research trial via the clinic's website, and if interested, could consent to participate and complete a brief online screening assessment, which took 10 to 15 min to complete. This assessment involved providing demographic, clinical and symptom data. Minimal inclusion and exclusion criteria were employed. Specifically, participants needed to: (1) aged 18 years or older; (2) have internet and device access; (3) self-report difficulties with anxiety or depression; (4) be able to read and understand English; and (5) not be at immediate risk of suicide. These criteria were all assessed via self-report in the screening assessment.

The trial was approved by the Macquarie University Medical Sciences Human Research Ethics Committee and prospectively registered on the Australian and New Zealand Clinical Trials Registry (ACTRN12621001554853).

### 2.2 Design

The current study was a two-arm parallel randomised control trial (RCT) with an immediate treatment group and a waitlist-control group. Eligible participants were automatically randomised using a 1:1 ratio at the completion of the initial assessment, ensuring allocation concealment. For operational efficiency, participants were enrolled in phases with new phases commencing every 8 weeks and start dates for phases published on the clinic website. After randomisation, a templated email was sent to participants confirming their randomisation and their start date for baseline assessment.

A primary reason for undertaking the current trial was to provide data concerning peoples' decision-making in taking up self-guided interventions (Fisher et al., 2024) and their levels of health literacy (Fisher et al., 2025), which have been reported in detail elsewhere. However, the current study reports the clinical outcomes of the trial, which have not reported in elsewhere.

### 2.3 The intervention

The study used an established transdiagnostic internet-delivered intervention, the Wellbeing Course, for adults anxiety and depression. The intervention has been evaluated in several large randomised controlled trials (Titov et al., 2013; Titov et al., 2015; Dear et al., 2015; Dear et al., 2016; Fogliati et al., 2016) and routine care settings (Titov et al., 2020; Hadjistavropoulos et al., 2022). The intervention comprises five core modules which are delivered over eight weeks and is based on transdiagnostic cognitive and behavioural principles. It is designed to be suitable for people with symptoms of anxiety and/or depression across the symptom severity range from mild to severe. The program content and format are described in detail elsewhere (Fisher et al., 2024; Fisher et al., 2025; Titov et al., 2015; Dear et al., 2015). In brief, the core modules provide psychoeducation while teaching psychological symptom formulation, thought challenging, controlled breathing, activity scheduling, activity pacing, graded exposure and lapse management. Several additional optional modules are made throughout the intervention, which cover topics like sleep management, problem solving, and assertive communication.

Participants can access the intervention via the clinic's website using a unique username and password. Each module is presented in the form of a slide show, comprising 30 to 40 slides and taking 10 to 20 min to read. The modules are released according to a schedule, which is designed support gradual progress. Each module comprises a combination of didactic information and case stories and examples from previous patients, sharing their experiences living with and learning to manage their symptoms. Modules are also accompanied by exercises for participants to implement the didactic information into their day-to-day life.

Importantly, no therapist contacts or support was provided before, during or after the intervention. However, if a significant deterioration in symptoms was detected a templated email was sent to the participant advising options for accessing urgent care and inviting the participant to contact the study therapist, if required. All participants completing the post-treatment questionnaires were also sent a templated email providing feedback about their symptoms at post-treatment.

### 2.4 Measures and outcomes

All data were collected via the eCentreClinic software platform, which presents relevant questionnaires to participants at login. Participants completed questionnaires at initial assessment, pre-treatment (weeks 1 to 2), mid-treatment (weeks 5 to 6) and post-treatment (weeks 9 to 11). Several questionnaires concerning health literacy, decisional conflict and participants casual attributions about their symptoms were also administered (Fisher et al., 2025). However, only the primary clinical measures are reported here.

The primary clinical outcomes were depression and anxiety, which were assessed the Patient Health Questionnaire 9-item (PHQ-9; score range 0 to 27) (Kroenke et al., 2001) and the Generalized Anxiety Disorder 7-Item (GAD-7; score range 0 to 21) (Spitzer et al., 2006), respectively. Scores on each measure are summed and total scores ≥10 on each measure is associated with a high probability of meeting diagnostic criteria for depressive or anxiety disorder, respectively. These outcomes were assessed at each time point. The Treatment Credibility and Expectancy Questionnaire (CEQ) (Devilly and Borkovec, 2000) was administered at pre-treatment. This measure comprises two subscales, each comprising 3 items, which are averaged to form an average treatment credibility score, and outcome expectancy score.

Three standardised questions were administered at post-treatment to assess participant satisfaction. These questions asked if they would “recommend the [course] to others?”, if the “[course] was worth [their] time?”, and “overall, how satisfied were [they]?”. Participants responded to the first two questions with a “yes” or “no” response, and the last using a 5-point scale from “very dissatisfied” to “very satisfied”.

Consistent with recommendations (Rozental et al., 2016), participants were asked at post-treatment whether they had noticed “a worsening in [their] mental health” since starting the intervention. If they responses “yes”, there were asked if they thought this worsening was due to the intervention.

### 2.5 Statistical analyses

Statistical analyses were conducted using R v4.3.1, the MICE v3.16.0 (Buuren and Groothuis-Oudshoorn, 2011) and geepack v1.3.9 (Halekoh et al., 2006) packages. The level of statistical significance was set at 0.05 for all analyses.

Descriptive statistics were calculated for participant demographic and baseline clinical characteristics. An intent-to-treat approach was adopted for all outcome analyses. Missing data was handled through the Multiple Imputation procedure with the generation of 5 modelled datasets, considering module completion, group, time, baseline severity, treatment credibility and outcome expectancies, consistent with past research (Karin et al., 2018; Dear, 2025).

Generalized estimating equation (GEE) models were used to examine symptom change over time, employing an unstructured working correlation matrix and a gamma log-link function. These models considered group and time as factors as well as their interaction. Separate models were run examining change in the overall sample, the clinical subsamples (i.e., defined as those scoring ≥10 on either the PHQ-9 or GAD-7) and among the treatment engaged subsample (i.e., defined as completing ≥4 of the 5 core modules). The clinical subgroup analyses were undertaken to examine change among patients who had clinically relevant symptoms and might be more expected to benefit from treatment. The treatment completer analyses were undertaken to examine change among patients who engaged with the treatment as intended. These subgroup analyses were deemed important as the overall analyses were expected to include a meaningful proportion of participants without meaningful symptoms on at least one outcome and to include many people who did not engage with treatment.

Power was estimated using a Monte Carlo simulation of 5000 synthetic datasets generated from a Gamma-log-link GEE with parameters based on prior PHQ-9 and GAD-7 data (φ = 0.20, ρ = 0.35). With 140 participants per group, the study would provide 0.80 power (α = 0.05, two-tailed) to detect a 15 % additional reduction in mean PHQ-9 or GAD-7 scores in the treatment group relative to control. However, it was planned to recruit more to adjust for attrition.

Consistent with past research (Karyotaki et al., 2017; Dear et al., 2022), the proportions of participants reporting ≥30 % and ≥50 % improvement from pre-treatment to post-treatment in anxiety and depression were calculated. Patients improving by ≥30 % were considered to have made a meaningful change and patients improving by ≥50 % were considered to have made a major clinical change. The proportions of participants making changes were analysed using a series of binary logistic regression. These analyses were also undertaken for the overall sample, the clinical subgroup, and for those completing treatment. To aid with interpreting the findings, these proportions were used to calculate the numbers needed to treat (NNT) metrics. NNTs were calculated using the formulae: NNT = 1/ARR, where the ARR is calculated as the event rate in the treatment group minus the event rate in the control group. The NNT represents the number of people who need to be provided the treatment to obtain one additional improved case over control.

## 3 Results

### 3.1 Inclusion, attrition and adherence

**Fig. 1:**
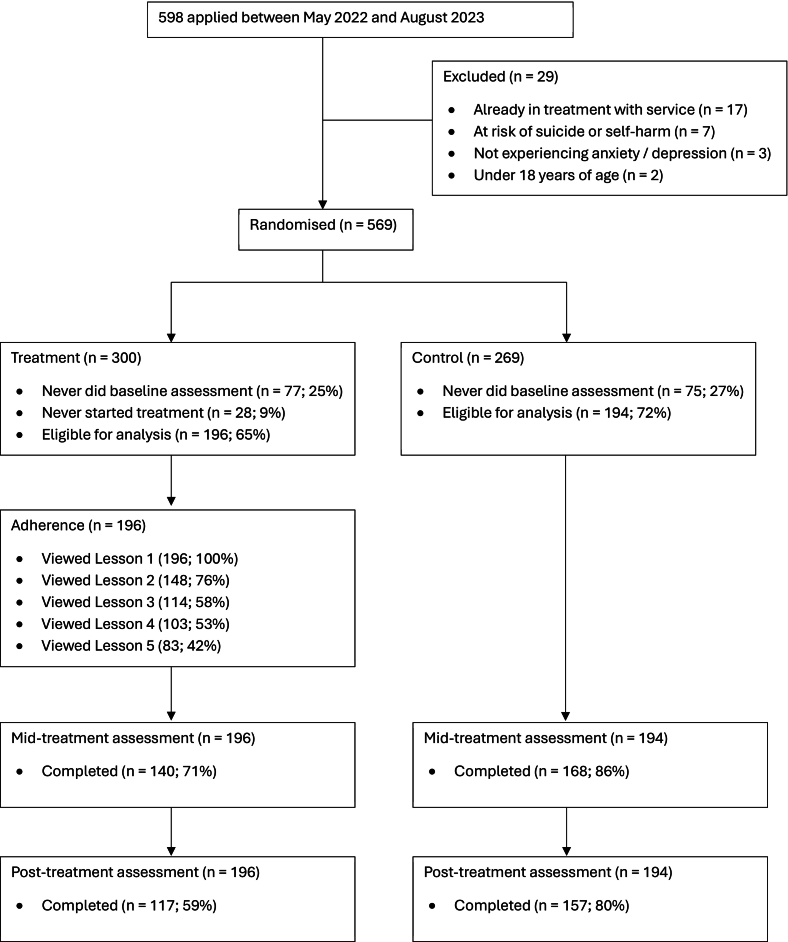
CONSORT flow chart.

Only 29/598 (4 %) people were excluded from the trial. The most common reason (17/29; 58 %) was participants already being engaged in another trial with the research clinic. Following randomisation, 77/300 (25 %) and 75/269 (27 %) participants in treatment and control did not complete their baseline assessments. The median number of days between completing an assessment and commencing baseline assessment was 13 days (M = 20; SD = 14). After baseline assessment, 28/300 (9 %) treatment group participants never started treatment and were excluded from analysis. A total of 117/196 (59 %) treatment group participants provided data at post-treatment, and 157/194 (80 %) control group participants provided post-treatment data. A total of 103/196 (53 %) of participants completed at least 4 of the 5 core intervention models, the threshold used to identify ‘treatment completers’. See Fig. 1 for detailed information of participant flow through the trial.

### 3.2 Patient characteristics

Patient demographic and clinical characteristics are reported in Table 1. Key attributes of the sample include that participants were mostly female, in a married or de facto relationship, and living in a capital city or its surrounding suburbs. Around half reported a university education and being in either full-time or part-time employment. Slightly less than half were currently taking medications for their mental health, with almost three quarters having taken them in the past. Similar proportions have previously received psychological treatment, and around a quarter were currently receiving psychological treatment. Around 4 % reported suicide-related thoughts in the past 7 days and 12 % reported having presented to an emergency department related to their mental health in the past.

**Table 1:** Participant demographic and clinical characteristics.

	Overall	Control	Treatment
n	390	194	196
Age (*M, SD)*	43.3 (12.4)	44 (12)	41 (12)
Gender (female: n, %)	342 (87 %)	168 (86 %)	174 (88 %)
Country (Australia: n, %)	363 (93 %)	181 (93 %)	182 (92 %)
Indigenous (yes: n, %)	10 (2 %)	7 (3 %)	3 (1 %)
Culturally and linguistically diverse (yes: n, %)	65 (16 %)	36 (18 %)	29 (14 %)
Relationship status (n. %)			
Single/dating	101 (25 %)	43 (22 %)	58 (29 %)
Married/de facto	235 (60 %)	127 (65 %)	108 (55 %)
Divorced/separated/widowed	42 (10 %)	20 (10 %)	22 (11 %)
Other	12 (3 %)	4 (2 %)	8 (4 %)
Education (n, %)			
High school or less	64 (16 %)	32 (16 %)	32 (16 %)
Trade certificate/Diploma	89 (22 %)	53 (27 %)	36 (18 %)
Bachelors degree	166 (42 %)	76 (39 %)	90 (45 %)
Masters or doctoral degree	71 (18 %)	33 (17 %)	38 (19 %)
Region (n, %)			
Capital city or surrounds	265 (67 %)	129 (66 %)	136 (69 %)
Other urban region	75 (19 %)	42 (21 %)	33 (16 %)
Rural or remote region	50 (12 %)	23 (11 %)	27 (13 %)
Vocational status (n, %)			
Full-time paid	147 (37 %)	73 (37 %)	74 (37 %)
Part-time paid	87 (22 %)	41 (21 %)	46 (23 %)
Student	26 (6 %)	14 (7 %)	12 (6 %)
At home parent	24 (6 %)	12 (6 %)	12 (6 %)
Unemployed	20 (5 %)	10 (5 %)	10 (5 %)
Registered sick or disabled	16 (4 %)	5 (2 %)	11 (5 %)
Retired	23 (5 %)	15 (7 %)	8 (4 %)
Suicide related thoughts in past 7 days (yes: n, %)	19 (4 %)	11 (5 %)	8 (4 %)
Ever called a mental health crisis service (yes: n, %)	87 (22 %)	42 (21 %)	45 (23 %)
Every presented to an Emergency Department for mental health (yes: n, %)	48 (12 %)	16 (8 %)	32 (16 %)
Mental Health Medications (yes: n, %)			
Ever	276 (70 %)	138 (71 %)	138 (70 %)
Currently	174 (44 %)	88 (45 %)	86 (43 %)
Psychological treatment (yes; n, %)			
Ever	297 (76 %)	146 (75 %)	151 (77 %)
Currently	104 (26 %)	50 (25 %)	54 (27 %)
Anxiety baseline symptom severity (n, %)^a^			
None	37 (9 %)	21 (10 %)	16 (8 %)
Mild	131 (33 %)	67 (34 %)	64 (32 %)
Moderate	109 (27 %)	52 (26 %)	57 (29 %)
Severe	113 (30 %)	54 (27 %)	59 (30 %)
Depression baseline symptom severity (n, %)^b^			
None	24 (6 %)	7 (3 %)	17 (8 %)
Mild	121 (31 %)	66 (34 %)	55 (28 %)
Moderate	119 (30 %)	62 (31 %)	57 (29 %)
Moderately severe	85 (21 %)	39 (20 %)	46 (23 %)
Severe	41 (10 %)	20 (10 %)	21 (10 %)
Duration of anxiety difficulties (n, %)			
< 2 weeks	1 (0 %)	0 (0 %)	1 (0 %)
2 weeks to 6 months	24 (6 %)	12 (6 %)	12 (6 %)
6 to 12 months	31 (7 %)	16 (8 %)	15 (7 %)
1 to 5 years	105 (26 %)	52 (26 %)	53 (27 %)
6 to 10 years	48 (12 %)	23 (11 %)	25 (12 %)
More than 10 years	157 (40 %)	77 (39 %)	80 (40 %)
Duration of depression difficulties (n, %)			
< 2 weeks	3 (0 %)	1 (0 %)	2 (1 %)
2 weeks to 6 months	30 (7 %)	14 (7 %)	16 (8 %)
6 to 12 months	26 (6 %)	16 (8 %)	10 (5 %)
1 to 5 years	68 (17 %)	33 (17 %)	35 (17 %)
6 to 10 years	36 (9 %)	16 (8 %)	20 (10 %)
More than 10 years	107 (27 %)	61 (31 %)	46 (23 %)
Perceived credibility and expectancy (*M, SD)*^c^			
Average credibility rating (1 to 9)	6.3 (1.4)	6.3 (1.4)	6.3 (1.4)
Average expectancy rating (0 to 10)	4.9 (2.0)	5.0 (2.1)	4.9 (1.9)

*Note.* All numbers reported at nearest whole number without rounding.

a Assessed using the PHQ-9.

b Assessed using the GAD-7.

c Assessed using the CEQ.

### 3.3 Patient satisfaction

Most respondents were either ‘very satisfied’ (31 %; 95 % CI: 23, 40) or ‘satisfied’ (44 %; 95 % CI: 34, 54) with the intervention overall. The remaining reported being ‘neutral’ (19 %; 95 % CI: 13, 29), ‘dissatisfied’ (3 %; 95 % CI: 1, 10) or ‘very dissatisfied’ (0 %; 95 % CI: 0, 10).

### 3.4 Self-reported deteriorations

Twelve percent (95 % CI: 7, 19) of participants reported worsening mental health symptoms during the intervention period. Only 2 % (95 % CI: 0, 7) reported the intervention was responsible for the worsening of their symptoms. Interestingly, all but one of these participants reported being ‘very satisfied’ with the intervention.

### 3.5 Depression symptoms

The means, standard deviations, percentage change and Hedge's g effect size estimates for depression symptoms in the overall sample and the two subsamples are shown in Table 2.

**Table 2:** Means, standard deviations and change statistics for depression symptoms (PHQ-9).

		M (SD)			% Change	Hedge's g	
	n	Pre	Mid	Post	Pre → Post	Pre → Post	Between at Post
Overall sample							
Treatment	196	12.1 (5.8)	10.5 (6.0)	9.5 (6.2)	21.4 % [13.8, 28.3]	0.43 [0.23, 0.63]	0.37 [0.17, 0.57]
Control	194	12.1 (5.3)	11.7 (5.7)	11.4 (5.6)	5.7 % [−1.2, 12.1]	0.07 [−0.13, 0.27]	
Clinical sample							
Treatment	124	15.6 (4.1)	13.2 (5.6)	12.1 (6.1)	22.4 % [15.4, 28.8]	0.67 [0.42, 0.93]	0.26 [0.01, 0.52]
Control	121	15.2 (4.2)	14.2 (5.1)	13.6 (5.2)	10.5 % [3.9, 16.4]	0.34 [0.08, 0.59]	
Engaged sample							
Treatment	103	11.5 (5.7)	9.5 (5.6)	8.0 (5.8)	30.5 % [20.0, 39.6]	0.61 [0.33, 0.89]	0.60 [0.35, 0.84]
Control	194	12.1 (5.3)	11.7 (5.7)	11.4 (5.6)	5.7 % [−1.1, 12.1]	0.13 [−0.07, 0.33]	

Note. Clinical sample = PHQ-9 ≥ 10; Engaged sample completed ≥4 modules.

#### 3.5.1 Overall sample

A significant time effect (*χ2* = 49.4, *p* < 0.001) and a significant group by time interaction (*χ2* = 17.9, *p* < 0.001) was observed for depression symptoms, indicating that the treatment group changed more over time. Pairwise comparisons revealed no significant differences between the two groups at pre-treatment (*p* = 0.936), and significantly lower symptoms scores among the treatment group at post-treatment (*p* = 0.004). They also revealed both the treatment group (*p* < 0.001), and the control group (*p* = 0.022), improved significantly from pre-treatment to post-treatment.

#### 3.5.2 Clinical subsample (scoring above clinical cut-offs)

Consistent with the overall sample analyses, a significant time effect (*χ2* = 55.0, *p* < 0.001) and a significant group by time interaction (*χ2* = 20.8, *p* < 0.001) was also observed. Pairwise comparisons revealed no significant differences between the two groups at pre-treatment (*p* = 0.347), and significantly lower symptoms scores among the treatment group at post-treatment (*p* = 0.032). They also revealed both the treatment group (*p* < 0.001), and the control group (*p* = 0.005), improved significantly from pre-treatment to post-treatment.

#### 3.5.3 Engaged subsample (completing ≥4/5 modules)

Also consistent with the overall sample analyses, a significant time effect (*χ2* = 36.5, *p* < 0.001) and a significant group by time interaction (*χ2* = 25.0, *p* < 0.001) was also observed. Pairwise comparisons revealed no significant differences between the two groups at pre-treatment (*p* = 0.402), and significantly lower symptoms scores among the treatment group at post-treatment (*p <* 0.001). They also revealed both the treatment group (*p* < 0.001), and the control group (*p* = 0.022), improved significantly from pre-treatment to post-treatment. Importantly, as expected, the proportion of change observed for those who engaged with treatment appears larger than observed for the overall sample.

### 3.6 Anxiety symptoms

The means, standard deviations, percentage change and Hedge's g effect size estimates for anxiety symptoms in the overall sample and the two subsamples are shown in Table 3.

**Table 3:** Means, standard deviations and change statistics for anxiety symptoms (GAD-7).

		M (SD)			% Change	Hedge's g	
	n	Pre	Mid	Post	Pre → Post	Pre → Post	Between at Post
Overall sample							
Treatment	196	11.3 (5.2)	9.7 (5.2)	9.1 (5.5)	19.6 % [12.4, 26.1]	0.41 [0.21, 0.61]	0.15 [−0.05, 0.35]
Control	194	10.8 (5.3)	10.2 (5.4)	9.9 (5.1)	8.9 % [2.0, 15.3]	0.17 [−0.03, 0.37]	
Clinical sample							
Treatment	116	15.6 (4.0)	13.2 (5.5)	12.1 (5.9)	22.4 % [15.4, 28.8]	0.69 [0.43, 0.96]	0.27 [0.01, 0.54]
Control	106	15.2 (3.9)	14.2 (4.8)	13.6 (4.9)	10.5 % [3.9, 16.4]	0.36 [0.09, 0.63]	
Engaged sample							
Treatment	103	10.8 (5.3)	9.1 (5.2)	7.9 (5.3)	27.0 % [16.8, 35.8]	0.55 [0.27, 0.82]	0.39 [0.14, 0.63]
Control	194	10.8 (5.3)	10.2 (5.4)	9.9 (5.1)	8.9 % [2.0, 15.3]	0.17 [−0.03, 0.37]	

Note. Clinical sample = GAD-7 ≥ 10; Engaged sample completed ≥4 modules.

#### 3.6.1 Overall sample

A significant time effect (*χ2* = 72.6, *p* < 0.001) and a significant group by time interaction (*χ2* = 13.2, *p* = 0.002) was observed for anxiety symptoms, indicating that the treatment group changed more over time. Pairwise comparisons revealed no significant differences between the two groups at pre-treatment (*p* = 0.395) or at post-treatment (*p* = 0.351). They also revealed both the treatment group (*p* < 0.001), and the control group (*p* < 0.001), improved significantly from pre-treatment to post-treatment.

#### 3.6.2 Clinical subsample (scoring above clinical cut-offs)

Consistent with the overall sample analyses, a significant time effect (*χ2* = 90.5, *p* < 0.001) and a significant group by time interaction (*χ2* = 9.8, *p* = 0.015) was also observed, indicating the treatment group improved more over time. Pairwise comparisons revealed no significant differences between the two groups at pre-treatment (*p* = 0.978) or at post-treatment (*p* = 0.101). They also revealed both the treatment group (*p* < 0.001), and the control group (*p* < 0.001), improved significantly from pre-treatment to post-treatment.

#### 3.6.3 Engaged subsample (completing ≥4/5 modules)

Similarly, consistent with the overall sample analyses, a significant time effect (*χ2* = 55.1, *p* < 0.001) and a significant group by time interaction (*χ2* = 18.2, *p* < 0.001) was also observed. Pairwise comparisons revealed no significant differences between the two groups at pre-treatment (*p* = 0.922), and significantly lower symptoms scores among the treatment group at post-treatment (*p =* 0.003). They also revealed both the treatment group (*p* < 0.001), and the control group (*p* < 0.001), improved significantly from pre-treatment to post-treatment. Importantly, as expected and consistent with the findings for depression, the proportion of change observed for those who engaged with treatment appears larger than observed for the overall sample.

### 3.7 Clinically significant change

The proportions of participants meeting criteria for a clinically significant change, and the resultant NNT, are reported in Table 4.

**Table 4:** Proportions making clinically significant improvements.

	Depression						Anxiety					
	≥ 30 %			≥ 50 %			≥ 30 %			≥ 50 %		
	% [95 % CI]	p	NNT	% [95 % CI]	p	NNT	% [95 % CI]	p	NNT	% [95 % CI]	p	NNT
Overall sample												
Treatment	42 % [34, 50]	<.001	4.4	27 % [20, 36]	<.001	5.3	38 % [32, 46]	<.001	5.9	25 % [19, 31]	<.001	6.6
Control	19 % [14, 26]			8 % [5, 14]			22 % [16, 29]			9 % [5, 16]		
Clinical Sample												
Treatment	41 % [31, 52]	<.001	4.4	23 % [15, 34]	.002	7.0	41 % [32, 51]	.013	7.2	26 % [19, 36]	.007	7.1
Control	19 % [12, 29]			9 % [5, 17]			27 % [19, 38]			12 % [6, 24]		
Engaged Sample												
Treatment	52 % [41, 62]	<.001	3.0	37 % [28, 48]	<.001	3.4	48 % [38, 58]	<.001	3.8	32 % [23, 42]	<.001	4.4
Control	19 % [14, 26]			8 % [5, 14]			22 % [16, 29]			9 % [5, 16]		

Note. Clinical sample = PHQ-9 ≥ 10 or GAD-7 ≥10; Engaged sample completed ≥ 4 modules.

In the overall samples, significantly greater proportions of participants met criteria for clinically meaningful change (i.e., ≥ 30 % improvement) in the treatment groups for both anxiety and depression. In terms of depression symptoms, 42 % and 19 % met criteria for a clinically meaningful improvement in the treatment and control groups, respectively. Similarly, in terms of anxiety symptoms, 38 % and 22 % met criteria for clinically meaningful improvement in the treatment and control groups, respectively. The resultant NNTs for depression and anxiety symptoms were 4.4 and 5.9, respectively.

Similarly, in the overall samples, significantly greater proportions of treatment group participants also meet criteria for major clinically meaningful changes (i.e., ≥ 50 % improvement) in in both anxiety and depression. Specifically, 27 % and 8 % of treatment and control group participants made major changes in depression, respectively, with an NNT of 5.3. Similarly, 25 % and 9 % of treatment group and control group participants made major changes in anxiety, respectively, with an NNT of 6.6.

## 4 Discussion

The aim of the current trial was to evaluate the real-world effectiveness of an entirely self-guided, internet-delivered transdiagnostic psychological intervention for adults with anxiety and depression. The trial aimed to examine the intervention under real-world conditions and thus imposed minimal inclusion and exclusion criteria. In the current study, relatively high levels of treatment completion and satisfaction were observed considering the self-guided nature of the intervention, with 53 % completing ≥4 of the core modules and 75 % reporting being satisfied with the treatment. In the overall sample, significant improvements were observed in the treatment group, compared with control, to post-treatment in both anxiety (treatment = 19 %; control = 8 %) and depression (treatment = 21 %; control = 5 %). Similar change was observed for those with clinical level symptoms of anxiety and depression, while there was evidence of larger effects (% improvement) among those engaging for treatment with both anxiety (treatment = 27 %) and depression (treatment 30 %). These changes in symptoms also corresponded to significantly greater proportions of participants meeting criteria for clinically meaningful change (≥ 50 %) in the treatment group versus control group in both anxiety and depression, with NNTs of 6.6 and 5.3, respectively. These NNTs indicate that approximately 6 people needed to be provided treatment for one additional person to make a major clinical improvement.

The current study extends on the few available studies examining entirely self-guided transdiagnostic interventions (Batterham et al., 2021; Titov et al., 2013) and is significant in that it included few inclusion or exclusion criteria. It is noteworthy that most of these participants would be excluded from most randomised controlled trials, resulting in samples that look less like those seen in routine care (Wells, 1999; Rothwell, 2005). For example, more than 10 % of participants presented with severe depressive symptoms (i.e., PHQ-9 scores ≥20), approximately 4 % reported suicide-related thoughts in the past 7 days, 26 % reported concurrent engagement in psychological treatment, and 44 % were taking medications for their mental health. Thus, the estimates of effects from the current study are encouraging given the sample provided treatment.

Although the effects are modest, the current findings are encouraging for the real-world effectiveness of an entirely self-guided, internet-delivered transdiagnostic intervention for adults with anxiety and depression with minimal exclusion criteria. These findings are consistent with outcomes of meta-analytic studies of disorder-specific treatments for depression with, for example, an individual patient data meta-analysis of self-guided internet-delivered treatments obtaining a hedge's g effect size of 0.27 and an NNT of 8 for achieving ≥50 % change in depression symptoms (Karyotaki et al., 2017). Unfortunately, similar meta-analyses examining the effects of self-guided interventions for anxiety are not unavailable for comparison. The effects observed in the current study are smaller than those reported by recent meta-analyses of internet-delivered transdiagnostic interventions. For example, a recent meta-analysis (k = 42; *n* = 4982) found moderate effect sizes for both anxiety (g = 0.58; 95 % CI: 0.47, 0.69) and depression (g = 0.65; 95 % CI: 0.53, 0.77) over waitlist control groups (Liu et al., 2025). However, careful review of the included studies indicates that almost all involved some clinician contact, either before or during the intervention, potentially explaining the larger effects. Nevertheless, taken together, the current findings suggest that transdiagnostic self-guided internet-delivered interventions have meaningful public health potential, particularly given the lower intervention-related costs likely associated with self-guided treatments (Hedman et al., 2012; Dear et al., 2021). Their potential would seem highest for people for whom speaking with a clinician represents a barrier to care, and for contexts where the resources for clinician-guided intervention may simply not be available.

The current study observed significant improvements in anxiety symptoms in the control groups over time, which significantly reduced the absolute difference observed between treatment and control groups at post-treatment. This contrasts with depression symptoms where no significant change was observed among the control group. The reasons for this are unclear, however small improvements in anxiety in treatment groups have been observed in large meta-analytic studies (Scott et al., 2022). One possibility is that people seek treatment for anxiety following the onset of an acute stressor, which resolves over time. This effect might be particularly prominent in entirely self-guided treatments because of their accessibility and the low burden associated with their uptake. It could also be that the regular symptom assessment and the associated reflection on symptoms involved in the control condition may facilitated some coping that improved symptoms. Related to this, it is noteworthy that approximately 26 % of participants completed an initial screening assessment but never commenced baseline assessment or treatment. The reasons why such a meaningful proportion never continue with treatment, especially given the low commitment and burden associated with a self-guided treatment, have not been established. However, this level of drop-out before treatment is common in trials of internet-delivered treatment (Bisby et al., 2022; Linardon et al., 2025) and when these treatments are delivered in routine care (Etzelmueller et al., 2020; Titov et al., 2020), and examination of the reasons for non-commencement would be a valuable future direction for research, especially focused on self-guided treatments.

One final finding from the current study was that treatment engagement appeared associated with significantly greater treatment-related change, and higher proportions of patients meeting criteria for clinical change. For example, looking at anxiety symptoms, the treatment engaged sample reported improvements of 27 % [95 % CI: 16, 35] compared with 19 % [95 % CI: 12, 26]; a relative increase in the clinical effect of approximately 40 %. While caution is needed around these findings given uncertainty around the point estimates, this general finding is consistent with much of the available literature concerning adherence and symptom change in anxiety and depression (Karyotaki et al., 2017; Haller et al., 2023; Kazlauskas et al., 2020). It remains unclear whether adherence increases outcomes or people achieving better outcomes adhere more, or some reciprocal combination of the two. Nevertheless, the current study extends the findings of the current literature to indicate that adherence is also associated with better outcomes in entirely self-guided transdiagnostic interventions as well. Moreover, without commenting on the mechanism, it appears possible to conclude that people who engage with treatment appear to obtain better outcomes.

The findings of the current study should be considered in light of several limitations. First, there was a meaningful proportion of missing data at post-treatment, particularly among the treatment group (treatment = 41 %; control = 20 %). This was addressed through the use of conservative multiple imputation models informed by dedicated missing data research concerning internet-delivered interventions for anxiety and depression (Karin et al., 2018; Dear, 2025). Nevertheless, some caution is needed around the resultant estimates as a result. Second, only a limited number of clinical outcomes were assessed, and only basic demographic and clinical history data was collected. Third, longer-term outcomes were not assessed and so caution is needed around the maintenance of effects over time. Fourth, the outcomes for only two potentially important subsamples were explored; that is, participants with clinical level symptoms and those who engaged in treatment. Thus, the results of these analyses should be treated with some caution as an exhaustive evaluation of potential mediators of outcomes were not performed, and there may have been other factors more important for understanding treatment response. Fifth, the current study employed a well-established transdiagnostic intervention, which is not optimised for mobile access and involves considerable reading (e.g., 10 to 20 min per module) without any audiovisual content (Titov et al., 2015; Dear et al., 2015). Thus, while typical of many internet interventions (Andersson, 2025), it is possible that the format of the intervention also affected engagement and outcomes, particularly as increasingly more people are using mobile devices to access services and preferences for the consumption of content (e.g., more video, audio, brief text) are changing.

In summary, the current study suggests that entirely, self-guided transdiagnostic, internet-delivered interventions have potential for adults with symptoms of anxiety and depression. Significant changes in symptoms and clinically meaningful improvements in symptoms were observed, alongside relatively high levels of treatment engagement and treatment satisfaction. Future research exploring who is most likely to benefit from this form of treatment, and how it might be improved to enhance engagement and benefit is worthwhile. Nevertheless, this form of treatment would seem to have specific potential for people for whom speaking with a clinician represents a barrier to care, or contexts where the resources for clinician-guided intervention are not available.

## Declaration of competing interest

Professors Titov and Dear are authors and developers of the Wellbeing Course but derive no financial benefit or royalties.

## Data Availability

Data is available for validation purposes subject to appropriate Australian Human Research Ethics Committee approval and the establishment of an institutional data sharing agreement.

## References

[bb0005] Andersson G. (2025). What makes internet-based interventions work?. World Psychiatry.

[bb0010] Andersson G., Titov N., Dear B.F., Rozental A., Carlbring P. (2019). Internet-delivered psychological treatments: from innovation to implementation. World Psychiatry.

[bb0015] Andrews G., Basu A., Cuijpers P., Craske M.G., McEvoy P., English C.L. (2018). Computer therapy for the anxiety and depression disorders is effective, acceptable and practical health care: an updated meta-analysis. J. Anxiety Disord..

[bb0020] Batterham P.J., Calear A.L., Farrer L., Gulliver A., Kurz E. (2021). Efficacy of a transdiagnostic self-help internet intervention for reducing depression, anxiety, and suicidal ideation in adults: randomized controlled trial. J. Med. Internet Res..

[bb0025] Bisby M.A., Karin E., Hathway T., Scott A.J., Heriseanu A.I., Dudeney J. (2022). A meta-analytic review of randomized clinical trials of online treatments for anxiety: inclusion/exclusion criteria, uptake, adherence, dropout, and clinical outcomes. J. Anxiety Disord..

[bb0030] Buuren S.V., Groothuis-Oudshoorn K. (2011). mice: Multivariate imputation by chained equations in *R*. J. Stat. Softw..

[bb0035] Cuijpers P., Miguel C., Ciharova M., Ebert D., Harrer M., Karyotaki E. (2023). Transdiagnostic treatment of depression and anxiety: a meta-analysis. Psychol. Med..

[bb0040] Dalgleish T., Black M., Johnston D., Bevan A. (2020). Transdiagnostic approaches to mental health problems: current status and future directions. J. Consult. Clin. Psychol..

[bb0045] Dear B.F. (2025). Credibility and expectations: important factors for understanding clinical response, treatment completion, and dropout in internet-delivered psychological interventions. J. Consult. Clin. Psychol..

[bb0050] Dear B.F., Staples L.G., Terides M.D., Karin E., Zou J., Johnston L. (2015). Transdiagnostic versus disorder-specific and clinician-guided versus self-guided internet-delivered treatment for generalized anxiety disorder and comorbid disorders: a randomized controlled trial. J. Anxiety Disord..

[bb0055] Dear B.F., Staples L.G., Terides M.D., Fogliati V.J., Sheehan J., Johnston L. (2016). Transdiagnostic versus disorder-specific and clinician-guided versus self-guided internet-delivered treatment for social anxiety disorder and comorbid disorders: a randomized controlled trial. J. Anxiety Disord..

[bb0060] Dear B.F., Karin E., Fogliati R., Dudeney J., Nielssen O., Scott A.J. (2021). A cost-effectiveness analysis of an internet-delivered pain management program delivered with different levels of clinician support: results from a randomised controlled trial. J. Pain.

[bb0065] Dear B.F., Scott A.J., Fogliati R., Gandy M., Karin E., Dudeney J. (2022). The chronic conditions course: a randomised controlled trial of an internet-delivered transdiagnostic psychological intervention for people with chronic health conditions. Psychother. Psychosom..

[bb0070] Devilly G.J., Borkovec T.D. (2000). Psychometric properties of the credibility/expectancy questionnaire. J. Behav. Ther. Exp. Psychiatry.

[bb0075] Etzelmueller A., Vis C., Karyotaki E., Baumeister H., Titov N., Berking M. (2020). Effects of internet-based cognitive behavioral therapy in routine care for adults in treatment for depression and anxiety: systematic review and meta-analysis. J. Med. Internet Res..

[bb0080] Fisher A., Eugene Dit Rochesson S., Bisby M.A., Scott A.J., Gandy M., Heriseanu A. (2024). Uptake of a self-guided digital treatment for depression and anxiety: a qualitative study exploring patient perspectives and decision-making. Health Expect Int. J. Public Particip. Health Care Health Policy.

[bb0085] Fisher A., Bisby M., Hathway T., Rezwan A., Ayre J., Muscat D. (2025). Assessing health literacy and its impacts among people accessing unguided internet-delivered cognitive behaviour therapy for depression and anxiety: a treatment-seeking cohort study. Adv. Ment. Health.

[bb0090] Fogliati V.J., Dear B.F., Staples L.G., Terides M.D., Sheehan J., Johnston L. (2016). Disorder-specific versus transdiagnostic and clinician-guided versus self-guided internet-delivered treatment for panic disorder and comorbid disorders: a randomized controlled trial. J. Anxiety Disord..

[bb0095] Gorman J.M. (1996). Comorbid depression and anxiety spectrum disorders. Depress. Anxiety.

[bb0100] Hadjistavropoulos H.D., Peynenburg V., Thiessen D.L., Nugent M., Karin E., Staples L. (2022). Utilization, patient characteristics, and longitudinal improvements among patients from a provincially funded transdiagnostic internet-delivered cognitive behavioural therapy program: observational study of trends over 6 years. Can. J. Psychiatr..

[bb0105] Halekoh U., Højsgaard S., Yan J. (2006). The *R* package **geepack** for generalized estimating equations. J. Stat. Softw..

[bb0110] Haller K., Becker P., Niemeyer H., Boettcher J. (2023). Who benefits from guided internet-based interventions? A systematic review of predictors and moderators of treatment outcome. Internet Interv..

[bb0115] Hedman E., Ljótsson B., Lindefors N. (2012). Cognitive behavior therapy via the internet: a systematic review of applications, clinical efficacy and cost–effectiveness. Expert Rev. Pharmacoecon. Outcomes Res..

[bb0120] Hedman-Lagerlöf E., Carlbring P., Svärdman F., Riper H., Cuijpers P., Andersson G. (2023). Therapist-supported internet-based cognitive behaviour therapy yields similar effects as face-to-face therapy for psychiatric and somatic disorders: an updated systematic review and meta-analysis. World Psychiatry.

[bb0125] Karin E., Dear B.F., Heller G.Z., Crane M.F., Titov N. (2018). “Wish you were here”: examining characteristics, outcomes, and statistical solutions for missing cases in web-based psychotherapeutic trials. JMIR Ment. Health.

[bb0130] Karyotaki E., Riper H., Twisk J., Hoogendoorn A., Kleiboer A., Mira A. (2017). Efficacy of self-guided internet-based cognitive behavioral therapy in the treatment of depressive symptoms: a meta-analysis of individual participant data. JAMA Psychiatry.

[bb0135] Karyotaki E., Efthimiou O., Miguel C., Bermpohl F.M.G., Furukawa T.A., Cuijpers P. (2021). Internet-based cognitive behavioral therapy for depression: a systematic review and individual patient data network meta-analysis. JAMA Psychiat..

[bb0140] Kazdin A.E., Blase S.L. (2011). Rebooting psychotherapy research and practice to reduce the burden of mental illness. Perspect. Psychol. Sci..

[bb0145] Kazlauskas E., Eimontas J., Olff M., Zelviene P., Andersson G. (2020). Adherence predictors in internet-delivered self-help intervention for life stressors-related adjustment disorder. Front. Psychol..

[bb0150] Kessler R.C., Petukhova M., Sampson N.A., Zaslavsky A.M., Wittchen H. (2012). Twelve-month and lifetime prevalence and lifetime morbid risk of anxiety and mood disorders in the United States. Int. J. Methods Psychiatr. Res..

[bb0155] Kolaas K., Berman A.H., Hedman-Lagerlöf E., Lindsäter E., Hybelius J., Axelsson E. (2024). Internet-delivered transdiagnostic psychological treatments for individuals with depression, anxiety or both: a systematic review with meta-analysis of randomised controlled trials. BMJ Open.

[bb0160] Krieger T., Bur O.T., Weber L., Wolf M., Berger T., Watzke B. (2023). Human contact in internet-based interventions for depression: a pre-registered replication and meta-analysis of randomized trials. Internet Interv..

[bb0165] Kroenke K., Spitzer R.L., Williams J.B.W. (2001). The PHQ-9: validity of a brief depression severity measure. J. Gen. Intern. Med..

[bb0170] Linardon J., Messer M., Reid R., Bolger T., Andersson G. (2025). Absolute and relative rates of treatment non-initiation, dropout, and attrition in internet-based and face-to-face cognitive-behavioral therapy: a meta-analysis of randomized controlled trials. Cogn. Behav. Ther..

[bb0175] Liu J., Li C., Qiu Y., Yu Y., Zeng L., Wu M. (2025). Efficacy of internet-delivered universal and tailored transdiagnostic interventions for anxiety and depression: a systematic review and meta-analysis of randomized controlled trials. Psychiatry Res..

[bb0180] Lorenzo-Luaces L., Zimmerman M., Cuijpers P. (2018). Are studies of psychotherapies for depression more or less generalizable than studies of antidepressants?. J. Affect. Disord..

[bb0185] Mojtabai R., Olfson M., Sampson N.A., Jin R., Druss B., Wang P.S. (2011). Barriers to mental health treatment: results from the National Comorbidity Survey Replication. Psychol. Med..

[bb0190] Rothwell P.M. (2005). External validity of randomised controlled trials: “to whom do the results of this trial apply?”. Lancet.

[bb0195] Rozental A., Kottorp A., Boettcher J., Andersson G., Carlbring P. (2016). Negative effects of psychological treatments: an exploratory factor analysis of the negative effects questionnaire for monitoring and reporting adverse and unwanted events. PLoS One.

[bb0200] Scott A.J., Bisby M.A., Heriseanu A.I., Hathway T., Karin E., Gandy M. (2022). Understanding the untreated course of anxiety disorders in treatment-seeking samples: a systematic review and meta-analysis. J. Anxiety Disord..

[bb0205] Spitzer R.L., Kroenke K., Williams J.B.W., Löwe B. (2006). A brief measure for assessing generalized anxiety disorder: the GAD-7. Arch. Intern. Med..

[bb0210] Titov N., Dear B.F., Johnston L., Lorian C., Zou J., Wootton B. (2013). Improving adherence and clinical outcomes in self-guided internet treatment for anxiety and depression: randomised controlled trial. PLoS One.

[bb0215] Titov N., Dear B.F., Staples L.G., Terides M.D., Karin E., Sheehan J. (2015). Disorder-specific versus transdiagnostic and clinician-guided versus self-guided treatment for major depressive disorder and comorbid anxiety disorders: a randomized controlled trial. J. Anxiety Disord..

[bb0220] Titov N., Dear B., Nielssen O., Staples L., Hadjistavropoulos H., Nugent M. (2018). ICBT in routine care: a descriptive analysis of successful clinics in five countries. Internet Interv..

[bb0225] Titov N., Dear B.F., Nielssen O., Wootton B., Kayrouz R., Karin E. (2020). User characteristics and outcomes from a national digital mental health service: an observational study of registrants of the Australian MindSpot clinic. Lancet Digit Health.

[bb0230] Wells K.B. (1999). Treatment research at the crossroads: the scientific interface of clinical trials and effectiveness research. Am. J. Psychiatry.

[bb0235] Whiteford H.A., Degenhardt L., Rehm J., Baxter A.J., Ferrari A.J., Erskine H.E. (2013). Global burden of disease attributable to mental and substance use disorders: findings from the Global Burden of Disease Study 2010. Lancet.

[bb0240] Zimmerman M., Balling C., Chelminski I., Dalrymple K. (2019). Have treatment studies of depression become even less generalizable? Applying the inclusion and exclusion criteria in placebo-controlled antidepressant efficacy trials published over 20 years to a clinical sample. Psychother. Psychosom..

